# Key predictors of undernutrition among children 6–59 months in the Buea Health District of the Southwest region of Cameroon: a cross sectional community-based survey

**DOI:** 10.1186/s40795-022-00646-0

**Published:** 2022-12-13

**Authors:** Andinwoh Betterdel Ngassa, Henry Dilonga Meriki, Clarence Mvalo Mbanga, Léonie Dapi Nzefa, Xikombiso Mbhenyane, Ayuk Betrand Tambe

**Affiliations:** 1 Department of Public Health and Hygiene, Faculty of Health Sciences, University of Buea, P.O. Box 063, Buea, Cameroon; 2 Clinton Health Access Innitiative, Cameroon office, 3rd Floor, Y-Building Rue 1775 Nouvelle Route Bastos, Yaounde, Cameroon; 3 Department of Social Work, Faculty of Social Sciences, Linnaeus University, Växjö, Sweden; 4 Division of Human Nutrition, Department of Global Health, Stellenbosch University, P.O. Box 241, Cape Town, South Africa

**Keywords:** Buea-Cameroon, Children under-five, Drinking water, Predictors, Prevalence, Poor dietary diversity, Undernutrition

## Abstract

**Background:**

According to the 2018 Demographic and Health Survey, undernutrition remains a public health problem among Cameroonian children under-five. This varies across the country, greatest in areas with ongoing humanitarian crisis, such as the Southwest region. However, data on the burden of undernutrition in the Southwest region is sparse. This study aimed to assess the prevalence and predictors of undernutrition among children under-five in the Buea health district of the Southwest region of Cameroon.

**Methods:**

This was a community based cross-sectional study of 321 children under-five/caretaker pairs, surveyed from households selected using multistage randomized sampling. Data were collected by trained data collectors, with the aid of a structured, pre-tested questionnaire that captured information on sociodemographic characteristics, food security, dietary diversity and anthropometric measurements. The weight, height/length and mid upper arm circumference (MUAC) were measured using standardized instruments. Stunting, Wasting and Underweight of children were calculated from Z-scores of Height-for-age (HAZ), Weight-for-height (WHZ) and Weight-for-age (WAZ) based on 2006 WHO standards. Data was analysed using SPSS version 27.0. Predictors of malnutrition were obtained using multivariate logistic regression, adjusting for potential confounders.

**Results:**

Overall, 31.8% (102/321) of the children were undernourished (26.5% stunted, 1.6% underweight, 3.7% wasted). Drinking water from inappropriate sources (OR: 2.32, 95% CI: 1.30–4.15) and a Dietary Diversity Score < 4 (OR: 2.59, 95%CI: 1.46–4.61) were independently associated with increased risk of stunting. Children of the male sex were more likely to be wasted than females (OR: 5.34, 95%CI: 1.09–26.14).

**Conclusion:**

Childhood undernutrition, particularly stunting is common in the Buea Health District. Risk factors of undernutrition identified are potentially modifiable, highlighting the need for nutrition specific and sensitive interventions to improve dietary diversity, and the need to improve access to safe drinking water, and educate caretakers on the importance of clean potable water, good sanitation and hygiene for the proper growth and development of their children.

## Introduction

Undernutrition is a public health problem worldwide, particularly among children under-five. Globally in 2020, about 149.2 million (22% of children under-five) and 45.4 million (6.7% of children under-five) were estimated to be stunted and wasted respectively [1]. Undernutrition equally accounts for about 3.1 million deaths (45% of all deaths) among children under-five annually [2]. This burden, however, is disproportionately borne by the African continent. In effect, two out of five (41% or 61.4 million children) stunted children and more than a quarter (27% or 12.1 million children) of all wasted children under-five live in the African continent [1]. This burden varied across the country, greatest in rural localities and areas with ongoing humanitarian crisis [3].

The effects of undernutrition on the health of affected children are multiple, ranging from poor cognitive and physical development, to increased susceptibility to infections resulting from a diminished immune response [4, 5]. As such, knowledge on the factors that predispose children to undernutrition becomes key in the design and implementation of interventions targeting the condition. Several factors have been shown to negatively influence the dietary intake and consequently the nutritional status of children under-five. These range from age, gender, disease states, and genetic makeup, to socioeconomic and socio-cultural factors such as poverty, level of education, household size, employment status and religion [6].

In Cameroon, according to data from the 2018 Demographic and Health Survey (DHS), 11% of Cameroonian children under-five were underweight, 4% were wasted and 29% were stunted [3]. For over half a decade now, the Northwest and Southwest Regions have been plagued by a socio-political turned armed conflict that has negatively impacted the population by limiting access to food, medical care and water and sanitation amenities. This has predisposed the population particularly the more vulnerable groups such as children under-five, to health and nutrition related problems like anaemia, diarrhoea, parasitic infections, malaria, typhoid, and undernutrition [7]. However, data on undernutrition and on the factors predisposing to the condition in children under-five in the Buea health district are sparse. It is with the intention of filling this knowledge gap, that we set out to assess, using a community-based survey, the prevalence and risk factors of undernutrition amongst children under-five in the Buea health district of the Southwest Region of Cameroon.

Data from this study will inform interventions against childhood undernutrition designed by both local and international organizations, as well as the state, to be scaled as per the burden of the condition, and target those with the most need. Such interventions would help curb the burden of childhood undernutrition in Buea particularly and Cameroon at large and help achieve nutrition-related Sustainable Development Goals (SDGs).

## Methods

### Study design and setting

This was a community-based cross-sectional study, carried out over the 6 months period between March and June 2021 in the Buea health district. Buea is the capital city of the Southwest Region of Cameroon, one of the two English speaking regions of the country, situated in the eastern slope of mount Cameroon. The town occupies a surface area of 7000 km^2^ with a population of approximately 1,481,433 inhabitants. Most inhabitants practice agriculture as the main economic activity. From a heath perspective, the Buea health district is one of the 18 health districts of the Southwest Region of Cameroon, consisting of 25 health facilities (including a regional hospital, serving as one of the two referral hospitals of the region) spread out over seven health areas (Bokova, Molyko, Muea, Bokwango, Buea Road, Bova and Buea Town) for an estimated population of 168,366 inhabitants. The main diseases among children in Buea are anaemia, diarrhoea, parasitic infections, malaria, typhoid, and undernutrition [7].

### Study population and participant selection

The study targeted children of both sexes between the ages of 6–59 months living within the Buea health district. Children were recruited into the study if there were aged 6–59 months and lived within the Buea health district, and there was at least one adult aged 18 years or more to provide consent. Excluded from the study were children with a health condition (e.g., lumbar scoliosis) that could falsify anthropometric measurements or children whose caretaker denied consent. Caretakers selected for interview were preferentially the mother. In case the mother was not available, the father or other adult (aunt, grandparent etc.) directly responsible for the child was interviewed.

### Sampling

#### Sample size

A minimum of 278 participants calculated using the formula: n = ${Z}^2\times \frac{P\left(1-P\right)}{d^2}\times k$ [8], were required for the study, where: n = minimum sample size, z = confidence value = 1.96 for a 95% confidence interval, *p* = estimated prevalence of childhood malnutrition from a study done in a similar crisis setting = 9.0%% [9], k = design effect = 2, d = error margin = 0.05 and 10% attrition added.

#### Sampling technique

Surveyed households were selected using a multi-stage sampling technique. First, the health areas that make up the Buea health district were considered, and three of them (Bova, Bokwango, Buea Road) were selected by simple random sampling. Next, geographically accessible households with children 6–59 months were identified and surveyed for each health area. In each selected household, the mother, father or any adult present in the house at the time of the survey was then interviewed. In the event where consent was denied from a household head/ caretaker, the data collectors continued to the next eligible household. The number of households surveyed in each selected health area was proportionate to the estimated number of eligible households within the health area. In case, an eligible households with 2 or more eligible children, one child was selected randomly by ballot.

### Data collection

#### Data collection tool

Data was collected via kobo collect on android phones, using a validated structured questionnaire designed as a kobo collect form. The questionnaire captured information on the socio-demographic characteristics of both the child and caretaker; water, sanitation and hygiene practices of participants and the household; dietary diversity of the children 24 hours prior to the survey using the dietary diversity questionnaire [10]; household food insecurity, assessed using the household food insecurity access scale (HFIAS) [11], a nine questions tool used to distinguish food insecure from food secure households, and to estimate the prevalence of household food insecurity**;** and the medical history of the children/caretakers with particular focus on chronic diseases such as HIV/AIDS that could influence their nutritional statuses. The questionnaire was pretested in the Molyko health area to ensure clarity of language, appropriateness, and sufficiency. This allowed for adjustments and corrections to be made as necessary before effectively beginning the data collection process.

#### Measurement of variables

##### Anthropometric parameters

Height was measured using a UNICEF height board to the nearest 0.1 cm, following standard procedures to ensure readings were accurate. Children aged 24 months and younger were measured lying down with infant’s head against the top of the headboard of the infantometer (recumbent length), while those older than 24 months old were measured standing up straight (height) with the child’s buttocks, shoulder blades, and heels together touching the back of the stadiometer. Weight was measured using a battery powered portable Seca 216 digital floor scale to the nearest 0.1 kg. At the beginning of each day, scales were calibrated with a standard 5 kg weight and validated as accurate before use. For children younger than 24 months or those older than 24 months who were unable to stand, tared weighing was done. For children 24 months or older who could stand still, the child was weighed alone. Mid upper arm circumference (MUAC) was measured using a colour coded MUAC tape to the nearest 0.1 cm following standardized procedures to ensure accuracy [12]. The height, weight and MUAC anthropometric components were standardized. All measurements were done twice by the same study personnel and the average taken. If the two measurements were not within 2 units (0.2 kg for weight and 0.2 cm for height and MUAC), the measurer was instructed to repeat the measurement until there were at least two measurements within 2 units.

##### Undernutrition

Stunting and underweight were defined as Length/height-for-age ≤ − 2 standard deviations (SD) of the median, and weight-for-age ≤ − 2 SD respectively. Wasting was defined as either a weight-for-height Z score ≤ − 2 SD or a MUAC ≤12.5 cm [2].

##### Dietary diversity

Food items consumed by the children 24 hours prior to the survey were recorded and grouped into the seven essential food groups for children as recommended by the World Health Organization (WHO) notably breast milk, cereals and tubers, legumes and nuts, dairy products, flesh foods (meats/fish/poultry), eggs, vitamin A-rich fruits and vegetables, other fruits and vegetables [13]. A child was considered to have consumed a particular food group if they consumed at least one food item from the food group. Each food group was scored 1 if consumed by the child and 0 if not. The dietary diversity score (DDS) was then computed for each child by adding up all the 1’s from the different food groups consumed by the child. The total DDS ranged from a minimum of 0 (the child consumed none of the food groups) to a maximum of 7 (the child consumed all the food groups). Children who consumed at least four of the seven food groups (DDS ≥ 4) were considered to meet the minimum dietary diversity requirements, while those with a DDS < 4 were on the other hand considered to have poor dietary diversity [13].

##### Household food insecurity

Household food insecurity (HFI) was assessed using the Household Food Insecurity Access Scale (HFIAS) for the 4 weeks period preceding the survey. To obtain the HFIAS score, the answer to each HFIAS question was coded as follows: If the respondents answer to a question was ‘no’, the answer to that question was coded as ‘0’. In case the respondents answer to a question was ‘yes’, the answer was coded based on the frequency reported by the respondent as 1 = Rarely (once or twice in the past 4 weeks), 2 = Sometimes (three to ten times in the past 4 weeks), 3 = Often (more than ten times in the past 4 weeks) [11]. The total HFIAS score was then obtained by summing the score to all the different questions. Consequently, the score ranged from a minimum of 0 (the answer to all questions was ‘no’) to a maximum of 27 (the answer to all questions was ‘yes, often’). Higher scores indicated higher levels of food insecurity and vice versa. HFI categories were then generated following previously defined guidelines [11]. HFI was classified into severely food insecure, moderate food insecure, mildly food insecure and food secure.

### Data management

**Fig. 1:**
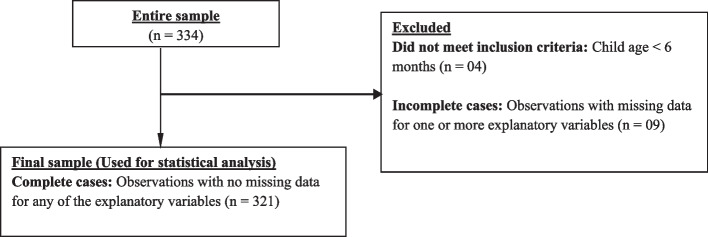
Sample selection process

The data was exported from the kobo collect platform as a Microsoft Excel spreadsheet, cleaned and analysed using STATA version 16.0 for Microsoft Windows. The initial sample consisted of 334 observations. Four (04) observations were deleted from the database as they did not correspond to the selection criteria (children aged below 06 months of age). The proportion of missing data for each explanatory variable varied from none to a maximum of 1.8%. As such, we assumed that the data were missing completely at random, and that deleting observations with the missing data did not yield a considerable change in the dataset. Hence, list-wise deletion was employed, with 09 observations dropped. A total of 321 observations with no missing data, were retained for use for statistical analysis (Fig. 1).

For the retained 321 observations, Z scores were generated using the ‘zanthro’ function of the STATA software, by comparing the recorded weight and height measurements of each child, to the WHO 2007 standard growth charts for children of the same sex and age [14]. Continuous variables were summarized as means with corresponding standard deviations, while categorical variables were presented as counts with percentages.

### Data analysis

The prevalence of each form of undernutrition (stunting, wasting and underweight) was compared between the different categories of each explanatory variable using the Chi square test or the fisher’s exact test as appropriate. Explanatory variables with *p* < 0.20 in the univariate analysis were retained for use as factors in multivariate analysis. The decision to use explanatory variables with *p* < 0.20 in the univariate analysis as factors in the multivariate model, was to maximize the chance of capturing variables that might influence the association studied or explain some of the variance in the outcome, even though they were not significantly associated to it. Multivariate logistic regression was used to determine characteristics independently associated with increased risk of stunting, underweight and wasting respectively. A 5% probability of a type I error was deemed acceptable. In all instances, two-sided *p* values were reported.

## Results

### Description of the study population

Table 1 summarizes the socio-demographic characteristics of the children, caretakers and households assessed during the survey. The age of the children ranged 6–59 months with an average of 33.6 ± 16.5 months. A greater proportion of the children were female (52.3%). Caretakers had a mean age of 30.1 ± 7.7 years and were mostly women (95.0%), with 84.4% of them attaining the secondary or tertiary level of education. Households had an average of 5.5 ± 2.4 persons (range: 02–15) children and adults inclusive. Majority of the households (60.4%) used unprotected/inappropriate drinking water sources and disposed of their waste appropriately (82.2%).

**Table 1:** Socio-demographic characteristics of the study sample

Children		Carers		Household	
Characteristic	n (%) *N* = 321	Characteristic	n (%) *N* = 321	Characteristic	n (%) *N* = 321
**Age category**		**Age category**		**Household size category**	
6–24 months	117 (36.4)	< 35 years	258 (80.4)	≤ 5 persons	187 (58.3)
25–59 months	204 (63.6)	36–55 years	60 (18.7)	> 5 persons	134 (41.7)
		> 55 years	03 (0.9)		
**Sex of child**		**Sex of carer**		**Decision maker**	
Female	168 (52.3)	Male	16 (5.0)	Father	25 (78.2)
Male	153 (47.7)	Female	305 (95.0)	Mother	67 (20.9)
				Other	03 (0.9)
**Chronic disease**		**BMI category**		**Other household member with chronic disease**	
Yes	01 (0.3)	Normal weight	89 (27.7)	Yes	26 (8.1)
No	320 (99.7)	Overweight	127 (39.6)	No	295 (91.9)
		Obese	105 (32.7)		
**Snacking between meals**		**Chronic disease**		**Water source**	
Yes	282 (87.9)	Yes	17 (5.3)	Protected**/**Appropriate	127 (39.6)
No	39 (12.1)	No	304 (94.7)	Unprotected/Inappropriate	194 (60.4)
**Number of meals daily**		**Smoking**		**Toilet type**	
1–2	18 (5.6)	Yes	05 (1.6)	Water closet	218 (67.9)
3–4	197 (61.2)	No	316 (98.4)	Pit toilet	103 (32.1)
> 4	106 (33.0)				
**Hand washing**		**Alcohol consumption**		**Sharing toilet**	
Soap and water always	56 (17.5)	Yes	110 (34.4)	Yes	49 (15.3)
Soap and water sometimes	235 (73.4)	No	170 (53.1)	No	272 (84.7)
Water only	29 (9.1)				
**Skipped a meal**		**Physical activity**		**Waste disposal**	
Yes	17 (5.3)	No exercise	111 (34.6)	Inappropriate	57 (17.8)
No	304 (94.7)	Once a week	24 (7.5)	Appropriate	264 (82.2)
		2–4 times a week	08 (2.5)		
		Daily	178 (55.4)		
		**Marital status**		**Household size category**	
		Single	78 (24.3)	≤ 5 persons	187 (58.3)
		Cohabiting	53 (16.5)	> 5 persons	134 (41.7)
		Married	190 (59.2)		
		**Employment status**			
		No job	99 (30.8)		
		Self-employed	175 (45.4)		
		Government / Private	47 (14.6)		
		**Educational level**			
		No schooling	6 (1.9%)		
		Primary	44 (13.7%)		
		Secondary	108 (33.6%)		
		University or more	163 (50.8%)		

*Freq* Frequency, *BMI* Body Mass Index

### Dietary diversity and food security

**Fig. 2:**
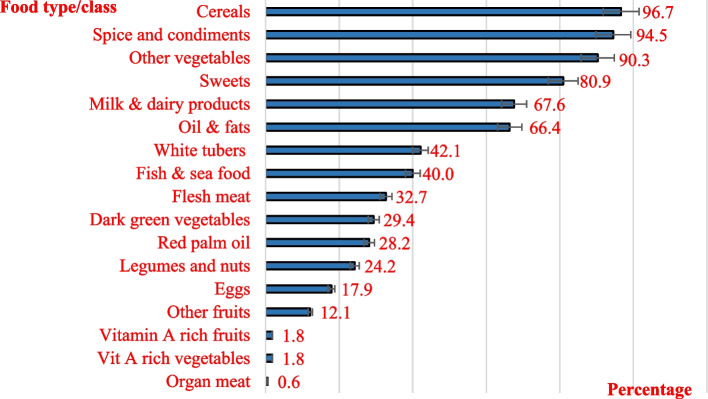
Consumption pattern of the different food groups by the surveyed children

The children consumed between 1 to 7 food groups with a mean DDS of 3.7 ± 1.0 food groups. The consumption pattern of the different food groups consumed by the children is depicted in Fig. 2. Cereals (96.7%) were the most consumed food group while vitamin A rich vegetables and fruits (1.8%) and organ meats (0.6%), were the least consumed (Fig. 2). The average HFIAS score was 7.2 ± 6.2 (range: 0–26 on a total of 27), with a median score of 7.0 [Interquartile range (IQR): 0–25].

### Prevalence of undernutrition

Of the 321 children retained in the final sample, 31.8% (102/321) were undernourished, being either stunted, wasted or underweight. Stunting was the most common form of undernutrition with a 26.5% (85/321). This prevalence was higher among children aged 6–24 months, those with poor dietary diversity (DDS < 4), and those whose caretakers were cohabiting or uneducated than others (Table 2). Stunting was equally significantly more prevalent among children from homes who drank water from inappropriate sources, used a pit toilet or shared toilets with other homes than others (Table 2). Wasting was the second most prevalent form of undernutrition reported in this study, with an overall prevalence of 3.7% (12/321). Male children and children consuming fewer meals a day were significantly more wasted (Table 3). Though statistically insignificant, the prevalence of wasting was equally greater among food insecure households (4.9%) compared to food secured households (2.4%) (Table 3). Underweight was the least common form of undernutrition in the sample, with an overall prevalence of 1.6% (05/321). This prevalence was significantly higher among girls than boys (Table 4). The prevalence of underweight was equally greater among children whose caretakers were obese, though borderline statistically insignificant (*p* = 0.05) (Table 4).

**Table 2:** Prevalence of stunting according to children, carer, and household characteristics

Children				Carers				Household			
Characteristic	N	Stunting (%)	***P***-value	Characteristic	N	Stunting (%)	***P***-value	Characteristic	N	Stunting (%)	***P***-value
**Age of child**			**0.03**^**a**^	**Age of carer**			0.22^a^	**Household size**			0.25^a^
6–24	117	33.3		< 35	258	28.3		≤ 5 persons	187	28.9	
25–59	204	22.6		> 55	03	33.3		> 5 persons	134	23.1	
				36–55	60	18.3					
**Sex of child**			0.10^a^	**Sex of Carer**			0.99^b^	**HFIAS**			0.28^a^
Male	153	30.7		Female	305	26.6		Severely food insecure	82	31.7	
Female	168	22.6		Male	16	25.0		Moderate food insecure	123	28.5	
								Mildly food insecure	32	25.0	
								Food secure	84	19.0	
**Snacking between meals**			0.61^a^	**BMI category**			0.98^a^	**Other household member**			
Yes	282	27.0		Normal weight	89	27.0		**with chronic disease**			0.61^a^
No	39	23.1		Overweight	127	26.8		Yes	26	30.8	
				Obese	105	25.7		No	295	26.1	
**Skipped meal**			0.57^b^	**Chronic disease**			0.26^b^	**Water source**			**0.001**^**a**^
No	304	27.0		No	304	27.3		Unprotected/Inappropriate	127	37.8	
Yes	17	17.7		Yes	17	11.8		Protected/Appropriate	194	19.1	
**Number of daily meals**			0.22^a^	**Smoking**			0.61^b^	**Toilet type**			**0.02**^**a**^
> 4	106	24.5		Yes	5	40.0		Pit toilet	103	35.0	
3–4	197	28.9		No	316	26.3		Flushing system	218	22.5	
1–2	18	11.1									
**Hand washing**			0.76^a^	**Alcohol consumption**			0.44^a^	**Sharing toilet**			**0.03**^**a**^
Water only	29	24.1		Yes	210	27.6		Yes	49	38.8	
Soap and water sometimes	235	27.7		No	110	23.6		No	272	24.3	
Soap and water always	56	23.2									
**Dietary diversity**			**0.001**^**b**^	**Physical activity**			0.33^b^	**Waste disposal**			0.76^a^
DDS < 4	112	37.5		Daily	178	26.4		Inappropriate	57	28.1	
DDS ≥ 4	209	20.6		2–4 times a week	08	25.0		Appropriate	264	26.1	
				Once a week	24	41.7					
				No exercise	111	23.4					
				**Marital status**			**0.04**^**a**^				
				Cohabiting	53	39.6					
				Married	190	25.3					
				Single	78	20.5					
				**Employment status**			0.12^a^				
				No job	99	26.3					
				Self-employed	175	29.7					
				Government / Private	47	14.9					
				**Educational level**			**0.01**^**b**^				
				No schooling	6	66.7					
				Primary	44	30.0					
				Secondary	108	33.3					
				University or more	163	19.6					

*N* Frequency, *DDS* Dietary Diversity Score, *BMI* Body Mass Index, *HFIAS* Household Food Insecurity Access Scale

^a^Chi square test

^b^Fisher’s exact test

**Table 3:** Prevalence of wasting according to children, carer, and household characteristics

Children				Carers				Household			
Characteristic	N	Wasting (%)	***P***-value	Characteristic	N	Wasting (%)	***P***-value^**a**^	Characteristic	N	Wasting (%)	***P***-value
**Age of child**			0.99^a^	**Age of carer**			0.99	**Household size**			0.99^a^
6–24	117	3.9		< 35	258	3.9		≤ 5 persons	187	3.7	
25–59	204	3.4		> 55	03	3.3		> 5 persons	134	3.7	
				36–55	60	0.0					
**Sex of child**			**0.05**^**b**^	**Sex of Carer**			0.99	**HFIAS**			0.86^b^
Male	153	5.9		Female	305	3.9		Severely food insecure	82	3.1	
Female	168	1.8		Male	16	0.0		Moderate food insecure	123	4.9	
								Mildly food insecure	32	3.7	
								Food secure	84	2.4	
**Snacking between meals**			0.65^a^	**BMI category**			0.21	**Other household member with chronic disease**			0.99^b^
Yes	282	5.1		Normal weight	89	4.5		Yes	26	3.9	
No	39	3.6		Overweight	127	5.7		No	295	3.7	
				Obese	105	1.6					
**Skipped meal**			0.49^a^	**Chronic disease**			0.49	**Water source**			0.87^a^
No	304	5.9		No	304	5.9		Unprotected/Inappropriate	127	3.9	
Yes	17	3.6		Yes	17	3.6		Protected/Appropriate	194	3.6	
**Number of daily meals**			**0.048**^**a**^	**Smoking**			0.17	**Toilet type**			0.93^b^
> 4	106	11.1		Yes	5	20.0		Pit toilet	103	3.9	
3–4	197	5.7		No	316	3.5		Flushing system	218	3.7	
1–2	18	2.0									
**Hand washing**			0.99^a^	**Alcohol consumption**			0.12	**Sharing toilet**			0.40^b^
Water only	29	3.6		Yes	210	6.4		Yes	49	6.1	
Soap and water sometimes	235	3.8		No	110	2.4		No	272	3.3	
Soap and water always	56	3.5									
**Dietary diversity**			0.76^a^	**Physical activity**			0.67	**Waste disposal**			0.45^b^
DDS < 4	112	4.5		Daily	178	5.4		Inappropriate	57	5.3	
DDS ≥ 4	209	3.4		2–4 times a week	08	0.0		Appropriate	264	3.4	
				Once a week	24	0.0					
				No exercise	111	3.4					
				**Marital status**			0.54				
				Cohabiting	53	3.9					
				Married	190	5.7					
				Single	78	3.2					
				**Employment status**			0.43				
				No job	99	4.0					
				Self-employed	175	4.6					
				Government / Private	47	0.0					
				**Educational level**			0.68				
				No schooling	6	0.0					
				Primary	44	2.3					
				Secondary	108	5.6					
				University or more	163	3.1					

*N* Frequency, *DDS* Dietary Diversity Score, *BMI* Body Mass Index, *HFIAS* Household Food Insecurity Access Scale

^a^Chi square test

^b^Fisher’s exact test

**Table 4:** Prevalence of underweight as per children, carer, and household characteristics

Children				Carers				Household			
Characteristic	N	UW (%)	***P***-value^*^	Characteristic	N	UW (%)	***P***-value^*^	Characteristic	N	UW (%)	***P***-value^*^
**Age of child**			0.66	**Age of carer**			0.99	**Household size**			0.17
6–24	117	0.9		< 35	258	1.6		≤ 5 persons	187	3.0	
25–59	204	2.0		> 55	03	1.7		> 5 persons	134	0.5	
				36–55	60	0.0					
**Sex of child**			**0.02**	**Sex of Carer**			0.99	**HFIAS**			
Male	153	0.0		Female	305	1.6		Severely food insecure	82	0.0	
Female	168	3.3		Male	16	0.0		Moderate food insecure	123	0.0	
								Mildly food insecure	32	1.6	
								Food secure	84	3.7	
**Snacking between meals**			0.99	**BMI category**			**0.05**	**Other household member with chronic disease**			0.35
Yes	282	1.8		Normal weight	89	1.1		Yes	26	3.9	
No	39	0.0		Overweight	127	0.0		No	295	1.4	
				Obese	105	3.8					
**Skipped meal**			0.99	**Chronic disease**			0.99	**Water source**			0.98
No	304	0.0		No	304	0.0		Unprotected/Inappropriate	127	1.6	
Yes	17	1.6		Yes	17	1.6		Protected/Appropriate	194	1.6	
**Number of daily meals**			0.20	**Smoking**			0.99	**Toilet type**			0.33
> 4	106	5.6		Yes	5	0.0		Pit toilet	103	2.9	
3–4	197	1.9		No	316	1.6		Flushing system	218	0.9	
1–2	18	1.0									
**Hand washing**			0.53	**Alcohol consumption**			**0.05**	**Sharing toilet**			0.17
Water only	29	3.5		Yes	210	3.6		Yes	49	4.1	
Soap and water sometimes	235	1.7		No	110	0.5		No	272	1.1	
Soap and water always	56	0.0									
**Dietary diversity**			0.35	**Physical activity**			0.99	**Waste disposal**			0.22
DDS < 4	112	2.7		Daily	178	1.8		Inappropriate	57	3.5	
DDS ≥ 4	209	1.0		2–4 times a week	08	0.0		Appropriate	264	1.1	
				Once a week	24	0.0					
				No exercise	111	1.7					
				**Marital status**			0.99				
				Cohabiting	53	1.9					
				Married	190	1.6					
				Single	78	1.3					
				**Employment status**			0.70				
				No job	99	1.0					
				Self-employed	175	2.3					
				Government / Private	47	0.0					
				**Educational level**			0.73				
				No schooling	6	0.0					
				Primary	44	2.3					
				Secondary	108	1.8					
				University or more	163	1.2					

*N* Frequency; *UW* Underweight, *DDS* Dietary Diversity Score, *BMI* Body Mass Index, *HFIAS* Household Food Insecurity Access Scale

^*^Fisher’s exact test

### Risk factors of undernutrition

Following multivariate logistic regression, the risk of stunting was greatest among children with poor dietary diversity (DDS < 4) (aOR: 2.59, CI: 1.46–4.61), and those from homes who drank water from inappropriate sources (aOR: 2.32, CI: 1.30–4.15) (Table 5). Wasting was more likely to be present among children of the male sex than female (aOR: 5.34, CI: 1.09–26.14) and children whose caretakers smoked (aOR: 61.59, CI: 3.42–1108.0) (Table 6). No factor was identified to be independently associated with a greater risk of being underweight (Table 7).

**Table 5:** Characteristics associated with stunting

Characteristic	N	Stunting (%)	OR (95% CI)	aOR (95% CI)	***P***-value
**Age of child**					0.82
6–24	117	33.3	1.72 (1.04; 2.85)	1.08 (0.54; 2.18)	
25–59	204	22.6	1	1	
**Sex of child**					0.07
Male	153	30.7	1.51 (0.92; 2.50)	1.69 (0.97; 2.95)	
Female	168	22.6	1	1	
**Marital status (carer)**					0.11
Cohabiting	53	39.6	2.54 (1.17; 5.54)	2.16 (0.91; 5.15)	
Married	190	25.3	1.31 (0.69; 2.48)	1.02 (0.49; 2.11)	
Single	78	20.5	1	1	
**Employment status (carer)**					0.30
No job	99	26.3	2.04 (0.81; 5.10)	0.67 (0.23; 2.02)	
Self-employed	22	40.9	2.42 (1.02; 5.74)	1.14 (0.42; 3.08)	
Government / Private employed	47	14.9	1	1	
**Educational level (carer)**					0.25
No schooling	6	66.7	8.19 (1.43; 46.69)	5.53 (0.90; 33.95)	
Primary	44	30.0	1.72 (0.81; 3.65)	1.22 (0.52; 2.86)	
Secondary	108	33.3	2.05 (1.17; 3.57)	1.47 (0.76; 2.82)	
University or more	163	19.6	1	1	
**Dietary diversity (child)**					**0.001**
DDS < 4	112	37.5	2.32 (1.39; 3.85)	**2.59 (1.46; 4.61)**	
DDS ≥ 4	209	20.6	1	1	
**Water source**					**0.004**
Unprotected/Inappropriate	127	37.8	2.58 (1.55; 4.28)	**2.32 (1.30; 4.15)**	
Protected/Appropriate	194	19.1	1	**1**	
**Toilet type**					0.69
Pit toilet	103	35.0	1.85 (1.11; 3.10)	1.18 (0.53; 2.62)	
Flushing system	218	22.5	1	1	
**Sharing toilet**					0.96
Yes	49	38.8	1.98 (1.04; 3.74)	1.02 (0.44; 2.36)	
No	272	24.3	1	1	

*N* Frequency, *aOR* Adjusted Odds ratio, *CI* Confidence Interval, *DDS* Dietary Diversity Score

**Table 6:** Characteristics associated with wasting

Characteristic	N	Wasting (%)	OR (95% CI)	aOR (95% CI)	***P***-value
**Sex of child**					**0.04**
Male	153	5.9	3.44 (0.91; 12.94)	**5.34 (1.09; 26.14)**	
Female	168	1.8	1	**1**	
**Number of daily meals**					0.08
1–2	18	11.1	6.03 (1.02; 35.49)	4.17 (1.04; 16.63)	
> 4	106	5.7	2.89 (0.80; 10.50)	4.17 (1.04; 16.63)	
3–4	197	2.0	1	1	
**BMI category (carer)**					0.32
Normal weight	89	4.5	2.94 (0.53; 16.42)	2.93 (0.47; 18.16)	
Obese	105	5.7	3.79 (0.75; 19.18)	3.63 (0.67; 19.70)	
Overweight	105	1.6	1	1	
**Smoking**					**0.005**
Yes	5	20.0	6.93 (0.71; 67.25)	**61.59 (3.42; 1108.0)**	
No	316	3.5	1	**1**	
**Alcohol consumption**					0.11
No	110	6.4	2.79 (0.86; 8.99)	2.92 (0.79; 10.73)	
Yes	210	2.4	1	1	

*N* Frequency, *aOR* Adjusted Odds ratio, *CI* Confidence Interval, *BMI* Body Mass Index

**Table 7:** Characteristics associated with underweight

Characteristic	N	Underweight (%)	OR (95% CI)	aOR (95% CI)	***P***-value
**Number of daily meals**					0.59
1–2	18	5.6	5.74 (0.49; 66.54)	4.01 (0.25; 63.17)	
> 4	106	1.9	1.88 (0.26; 13.50)	1.78 (0.23; 13.59)	
3–4	197	1.0	1	1	
**Alcohol consumption**					0.11
No	110	3.6	7.89 (0.87; 71.44)	6.34 (0.66; 61.19)	
Yes	210	0.5	1	1	
**Household size**					0.17
> 5 persons	134	3.0	5.72 (0.63; 51.79)	4.83 (0.51; 45.56)	
≤ 5 persons	187	0.5	1	1	
**Sharing toilet**					0.37
Yes	49	4.1	3.54 (0.62; 23.45)	2.58 (0.32; 20.64)	
No	272	1.1	1	1	

*N* Frequency, *aOR* Adjusted Odds ratio, *CI* Confidence Interval

## Discussion

This study sought to assess the prevalence and risk factors of undernutrition among children under-five in the Buea health district. We found prevalence’s of stunting, underweight and wasting of 26.5, 1.6 and 3.7% respectively, and found that inappropriate drinking water and a poor dietary diversity diet (DDS < 4) were independently associated with increased risk of stunting. Being male and having a smoking caretaker were linked with higher risks of being wasted.

The prevalence of stunting (26.5%) found among children under-five recorded in our study was higher when compared to similar to the national prevalence of 29% obtained during the 2018 DHS [3]. Although disease and inadequate food remain the major causes of malnutrition globally, lack of education, poor quality and inadequate health services, poverty and detrimental health practices add to these conditions in Cameroon. This prevalence is lower than those reported in other African and low-income countries [15, 16]. In a secondary analysis of data from 2960 children obtained from the Tanzania DHS of 2015–2016, the prevalence of stunting was estimated at 31% [15]. Results from a community based cross-sectional survey similar to ours in India indicated a prevalence of stunting as high as 45.7% [16], while Lawan et al*.* in Nigeria showed an even higher prevalence of stunting [17]. The discrepancies observed could be explained by the differences in the age group of children used in these studies. While our survey focused on children aged 6–59 months, the aforementioned studies focused on children aged 6–24 months. In addition, prevalence of wasting (3.7%) and underweight (1.6%) were low compared to a similar findings reported in Cameroon [18]. Children suffering from undernutrition have a weakened immune system, leaving them vulnerable to developmental delays, disease and death. The possible reason for this might be disparities among participants in socio-demographic characteristics, setting, wealth and access to health care.

Children drinking water from inappropriate sources were more likely to be stunted as compared to their peers drinking tap or mineral water. About 40% of children drank water from inappropriate sources, indicating that a good number of them were exposed to poor sanitation which could increase the risk of disease and undernutrition. The association between drinking water and stunting has been established by preceding authors [19–22]. The later statement is further strengthened by the UNICEF conceptual framework, which describes insufficient access to clean water, sanitation, and hygiene (WASH) as an underlying contributing factor to undernutrition [23]. Given its importance, access to appropriate, safe sources of drinking water was identified as a major public health problem and adopted as a human right by the United Nations general assembly in 2010. In effect, unsafe or contaminated water can lead to the transmission of diarrhoeal diseases such as cholera, dysentery, typhoid, and polio. Unsafe drinking water can equally lead to environmental enteropathies, which in the long-term result in undernutrition, anaemia, impaired brain development and growth stunting [24, 25].

Inadequate dietary diversity (DDS < 4) was found to be associated with higher odds of being stunted among children in the current study. Several studies have found a similar association [15–17, 26–28]. Dietary diversity is a good predictor of the dietary quality and micronutrient density in children [27, 29]. This is explained by the fact that overall, the consumption of animal-source foods like meat (consumed by 32.7% of children), fish (consumed by 40%), eggs (consumed by 18%) was poor among the children in our sample. Animal-source foods like meat, fish, milk, eggs, and poultry have a variety of micronutrients including vitamin A, vitamin B-12, riboflavin, calcium, iron, and zinc that are difficult to obtain in adequate quantities from plant sourced foods alone [30]. Insufficient intake of these nutrients may hinder the physical development of a child, resulting in stunting. This highlights dietary diversity as one of the important factors that could be targeted by policy makers and interventions to improve on the nutritional status of children in the Buea health district.

The prevalence of underweight and wasting stood at 1.6 and 3.7% respectively. This prevalence was lower than that recorded in most African and developing countries [15, 16]. The present study did not find an association between underweight or wasting and the dietary diversity of children. This is in line with findings from other studies [31, 32]. This might be due to the fact that underweight and particularly wasting are acute conditions resulting from shorter-term episodes of inadequate feeding or illnesses.

In this study, male children had a significantly higher risk of being wasted than their female counterparts. This association between the male sex and wasting has been reported in other African countries, notably Nigeria, and Ethiopia [33, 34]. A recent meta-analysis equally found the same association between the male sex and all three forms of undernutrition [35]. Though no direct scientific explanation to this association is known yet, a few attempts have been made to explain it and are worth mentioning. Boys have the tendency to engage in physical activities of much higher intensity thereby using up considerable amounts of energy meant for proper growth and development. On the other hand, girls are culturally expected to engage in less intense physical activity which includes staying home with their mothers near food preparation. As such they conserve and channel more energy to growth and development, and are therefore less likely to be malnourished.

## Limitations and strengths

Certain limitations should be considered when interpreting results obtained from this study. Given the cross-sectional design of the study, the direction of the associations observed cannot be ascertained. Conclusions on the causal effect of significant factors identified herein is therefore not possible. Also, a considerable portion of the data obtained was based on self-reported information provided by the caretakers. As a result, they could be a tendency for caretakers to under-report certain aspects such as their smoking status for instance, leading to social desirability bias. However, privacy was assured during data collection and participants provided their responses anonymously. As such there was little incentive to report inaccurate answers. Recall bias could equally come into play as caretakers may not properly recall all required information. Furthermore, certain indicators of the nutrition status like stunting represent a long-term cumulative process, whereas the dietary information available reflected dietary patterns 24 h prior to the survey. In addition, we were not able to attain the minimum sample size calculated a priori, thereby reducing our desired statistical power. However, we used robust statistical methods to analyse available data and remain confident in the results obtained and presented herein.

As strengths, we accounted for major risk factors and confounders of undernutrition in the analysis. Also, the probabilistic sampling method employed, implies that our sample could be reasonably representative of the population of children under five in the Buea health district. Thus, the findings of the current study could be generalised to setting with similar problem.

## Conclusion

Undernutrition, particularly stunting is prevalent among children under-five in the Buea health district. Poor dietary diversity and inappropriate drinking water were potentially modifiable risk factors of undernutrition identified. This highlights the need for both nutrition specific and sensitive interventions to improve dietary diversity, the access to safe drinking water and educate caretakers on the importance of water, sanitation and hygiene for the proper growth and healthy development of their children.

## Data Availability

The data set used in generating the results presented in this study is available from the corresponding author upon reasonable request.

## Abbreviations

aOR: Adjusted Odds Ratio
DDS: Dietary Diversity Score
DHS: Demographic and Health Survey
HFI: Household Food Insecurity
HFIAS: Household Food Insecurity Access Scale
IQR: Inter Quartile Range
MUAC: Mid Upper Arm Circumference
SD: Standard Deviation
SDG: Sustainable Development Goal
UNICEF: United Nations Children Fund
WASH: Water, Sanitation and Hygiene
WHO: World Health Organization

## References

[1] UNICEF / WHO / World Bank Group. Levels and Trends in Childhood malnutrition. Key findings of the 2021 edition. Geneva; 2021. Available from: https://www.who.int/publications/i/item/9789240025257

[2] Fact sheets - Malnutrition. Available from: https://www.who.int/news-room/fact-sheets/detail/malnutrition. [cited 7 Mar 2022].

[3] National Institute of Statistics (Cameroon) and ICF (2020). Cameroon demographic and health survey 2018.

[4] MAL-ED Network Investigators (2014). The MAL-ED study: a multinational and multidisciplinary approach to understand the relationship between enteric pathogens, malnutrition, gut physiology, physical growth, cognitive development, and immune responses in infants and children up to 2 years of age in resource-poor environments. Clin Infect Dis.

[5] Reinhardt K, Fanzo J (2014). Addressing chronic malnutrition through multi-sectoral, sustainable approaches: a review of the causes and consequences. Front Nutr..

[6] Akorimo R. Assessment of nutritional status and associated factors among children 6–59 months in Mpunge sub-county, Mukono district [Thesis]. Makerere University; 2019. Available from: http://dissertations.mak.ac.ug/handle/20.500.12281/7557. [cited 7 Mar 2022]

[7] Tesema GA, Worku MG, Tessema ZT, Teshale AB, Alem AZ, Yeshaw Y, Alamneh TS, Liyew AM (2021). Prevalence and determinants of severity levels of anemia among children aged 6–59 months in sub-Saharan Africa: a multilevel ordinal logistic regression analysis. PLoS One.

[8] Daniel WW, Cross CL (2018). Biostatistics: a foundation for analysis in the health sciences.

[9] Cumber SN, Jaila S, Nancy B, Tsoka-Gwegweni JM (2017). Under five malnutrition crises in the Boko haram area of Cameroon. South Afr J Clin Nutr.

[10] Kennedy G, Ballard T, Dop MC (2011). Guidelines for measuring household and individual dietary diversity.

[11] Coates J, Swindale A, Bilinsky P. Household Food Insecurity Access Scale (HFIAS) for Measurement of Food Access: Indicator Guide: Version 3: (576842013-001). Washington, DC: American Psychological Association; 2007. Available from: http://doi.apa.org/get-pe-doi.cfm?doi=10.1037/e576842013-001 [cited 15 Mar 2022].

[12] USAID, FANTA III, fhi 360 & PEPFAR (2016). Module 2: nutrition assessment and classification (NACS User's guide).

[13] World Health Organization (2008). Indicators for assessing infant and young child feeding practices [Part I: definition].

[14] WHO Multicentre Growth Reference Study Group (2006). WHO child growth standards based on length/height, weight and age. Acta Paediatr Suppl.

[15] Khamis AG, Mwanri AW, Ntwenya JE, Kreppel K (2019). The influence of dietary diversity on the nutritional status of children between 6 and 23 months of age in Tanzania. BMC Pediatr.

[16] Ahmad I, Khalique N, Khalil S, Urfi, Maroof M (2018). Dietary diversity and stunting among infants and young children: a Cross-sectional study in Aligarh. Indian. J Community Med.

[17] Lawan UM, Amole GT, Jahum MG, Sani A (2014). Age-appropriate feeding practices and nutritional status of infants attending child welfare clinic at a teaching Hospital in Nigeria. J Fam Community Med.

[18] Dapi Nzefa L, Monebenimp F, Äng C (2019). Undernutrition among children under five in the Bandja village of Cameroon, Africa. South Afr J Clin Nutr.

[19] Tette EMA, Sifah EK, Nartey ET (2015). Factors affecting malnutrition in children and the uptake of interventions to prevent the condition. BMC Pediatr.

[20] Belaynew W, Bantamen G (2014). Assessment of factors associated with malnutrition among under five years age children at Machakel Woreda, Northwest Ethiopia: a case control study. J Nutr Food Sci.

[21] Piniel A. Factors contributing to severe acute malnutrition among the under five children in Francistown-Botswana [Thesis]. Cape Town: University of the Western Cape; 2016. Available from: http://etd.uwc.ac.za/xmlui/handle/11394/5253 [cited 15 Mar 2022].

[22] Altare C, Delbiso TD, Mutwiri GM, Kopplow R, Guha-Sapir D (2016). Factors associated with stunting among pre-school children in southern highlands of Tanzania. J Trop Pediatr.

[23] United Nations Children’s Fund (2020). UNICEF conceptual framework on maternal and child nutrition.

[24] Ngure FM, Reid BM, Humphrey JH, Mbuya MN, Pelto G, Stoltzfus RJ (2014). Water, sanitation, and hygiene (WASH), environmental enteropathy, nutrition, and early child development: making the links. Ann N Y Acad Sci.

[25] John CC, Black MM, Nelson CA (2017). Neurodevelopment: the impact of nutrition and inflammation during early to middle childhood in low-resource settings. Pediatrics..

[26] Zongrone A, Winskell K, Menon P (2012). Infant and young child feeding practices and child undernutrition in Bangladesh: insights from nationally representative data. Public Health Nutr.

[27] Paudel R, Pradhan B, Wagle RR, Pahari DP, Onta SR (2012). Risk factors for stunting among children: a community based case control study in Nepal. Kathmandu Univ Med J.

[28] Tessema M, Belachew T, Ersino G (2013). Feeding patterns and stunting during early childhood in rural communities of Sidama, South Ethiopia. Pan Afr Med J.

[29] Nguyen PH, Avula R, Ruel MT, Saha KK, Ali D, Tran LM, Frongillo EA, Menon P, Rawat R (2013). Maternal and child dietary diversity are associated in Bangladesh, Vietnam, and Ethiopia. J Nutr.

[30] Zhang Z, Goldsmith PD, Winter-Nelson A (2016). The importance of animal source foods for nutrient sufficiency in the developing world: the Zambia scenario. Food Nutr Bull.

[31] Sié A, Tapsoba C, Dah C, Ouermi L, Zabre P, Bärnighausen T (2018). Dietary diversity and nutritional status among children in rural Burkina Faso. Int Health.

[32] Jones AD, Ickes SB, Smith LE, Mbuya MNN, Chasekwa B, Heidkamp RA (2014). World Health Organization infant and young child feeding indicators and their associations with child anthropometry: a synthesis of recent findings. Matern Child Nutr.

[33] Akombi BJ, Agho KE, Merom D, Hall JJ, Renzaho AM (2017). Multilevel analysis of factors associated with wasting and underweight among children under-five years in Nigeria. Nutrients..

[34] Dabale G, Sharma MK, Department of Statistics, Arba Minch University, P.O. Box: 21, Arba Minch, Ethiopia (2014). Determinants of wasting among under-five children in Ethiopia: (a multilevel logistic regression model approach). Int J Stats Med Res.

[35] Thurstans S, Opondo C, Seal A, Wells J, Khara T, Dolan C (2020). Boys are more likely to be undernourished than girls: a systematic review and meta-analysis of sex differences in undernutrition. BMJ Glob Health.

